# Genetic variation in histone modifications and gene expression identifies regulatory variants in the mammary gland of cattle

**DOI:** 10.1186/s12864-022-09002-9

**Published:** 2022-12-08

**Authors:** Claire P. Prowse-Wilkins, Thomas J. Lopdell, Ruidong Xiang, Christy J. Vander Jagt, Mathew D. Littlejohn, Amanda J. Chamberlain, Michael E. Goddard

**Affiliations:** 1 Agriculture Victoria, AgriBio, Centre for AgriBioscience, 5 Ring Road, Bundoora, Victoria 3082 Australia; 2 Faculty of Veterinary & Agricultural Science, University of Melbourne, Parkville, Victoria 3010 Australia; 3 Research and Development, Livestock Improvement Corporation, Private Bag 3016, Hamilton, 3240 New Zealand

**Keywords:** bovine, ChIP-seq, histone modifications, function, causal variants, allele specific QTL, molecular QTL, regulatory variants

## Abstract

**Background:**

Causal variants for complex traits, such as eQTL are often found in non-coding regions of the genome, where they are hypothesised to influence phenotypes by regulating gene expression. Many regulatory regions are marked by histone modifications, which can be assayed by chromatin immunoprecipitation followed by sequencing (ChIP-seq). Sequence reads from ChIP-seq form peaks at putative regulatory regions, which may reflect the amount of regulatory activity at this region. Therefore, eQTL which are also associated with differences in histone modifications are excellent candidate causal variants.

**Results:**

We assayed the histone modifications H3K4Me3, H3K4Me1 and H3K27ac and mRNA in the mammary gland of up to 400 animals. We identified QTL for peak height (histone QTL), exon expression (eeQTL), allele specific expression (aseQTL) and allele specific binding (asbQTL). By intersecting these results, we identify variants which may influence gene expression by altering regulatory regions of the genome, and may be causal variants for other traits. Lastly, we find that these variants are found in putative transcription factor binding sites, identifying a mechanism for the effect of many eQTL.

**Conclusions:**

We find that allele specific and traditional QTL analysis often identify the same genetic variants and provide evidence that many eQTL are regulatory variants which alter activity at regulatory regions of the bovine genome. Our work provides methodological and biological updates on how regulatory mechanisms interplay at multi-omics levels.

**Supplementary Information:**

The online version contains supplementary material available at 10.1186/s12864-022-09002-9.

## Introduction

Gene expression QTL studies seek to identify causal variants by finding genomic variants associated with differences in gene expression [1]. Gene expression is in itself an interesting complex trait, but it is also important because changes in gene expression might be the mechanism by which variants affect other complex traits such as fertility or disease susceptibility [2]. A complication of association studies is that the variants identified may be associated with the trait only because of linkage disequilibrium (LD) with the causal variant, not because the identified variant is causal [3]. However, there are numerous examples of disease and trait causing variants located in non-coding regions of the genome which are putatively functional [4, 5] where they are thought to be altering regulatory regions controlling gene expression. Therefore, one way to pinpoint causal variants for gene expression and other complex traits is to focus on expression QTL (eQTL) found in functional regions.

Functional regions have been found to be marked by modifications to histone proteins which form the nucleosome and are involved in packaging DNA in the nucleus of the cell [6]. For example, tri-methylation of histone H3 at its 4th lysine (H3K4Me3) is commonly found at promoters, mono-methylation at the same lysine (H3K4Me1) is found at enhancers and promoters, and acetylation of the 27th lysine (H3K27ac) is found at active regions of the genome [7]. Modified histones can be identified using chromatin immunoprecipitation followed by sequencing (ChIP-seq). This method uses antibodies to pull out genome regions marked by histone modifications and sequences them. The DNA sequence is mapped to the genome where it forms peaks at regions where the histone modifications were found [8]. Multiple studies have shown that eQTL are enriched in histone modification ChIP-seq peaks [9, 10]. However, histone modifications can be found in a large proportion of the genome so there may still be millions of variants in these regions [11]. Consequently, an additional filter is needed to find the variants that might be causal.

Studies have shown that gene expression level and histone modification peak height are correlated [12–14]. Therefore, one explanation for the enrichment of eQTL under histone modification ChIP-seq peaks is that these SNPs alter regulatory regions (as measured by histone modifications) which subsequently affects gene expression. Hence, an eQTL variant that is also altering histone modifications is an excellent candidate causal variant for gene expression and other complex traits. Variants affecting histone modifications (hQTL) can be identified by associating SNPs with differences in ChIP-seq peak height in the same way that variants affecting gene expression (eQTL) can be identified by associating SNPs with differences in gene expression [15]. eQTL and hQTL are classified as acting in *cis* or *trans*, where *cis* means that the allele on one homologous chromosome affects a feature (gene/peak) from that chromosome but not from the other homologous chromosome. A QTL in *trans* means that an allele can affect a feature on any chromosome [16]. In practice, variants near the feature are usually assumed to be acting in *cis* [17].

Variants acting in *cis* can also be detected because they cause allele specific expression (ASE) or allele specific binding (ASB). That is, one allele from the gene is expressed more (ASE) or one allele is marked more often by a functional marker (ASB). QTL analysis using ASB or ASE is statistically independent of analysis from traditional eQTL and hQTL analyses, because it relies on comparisons within an individual not between individuals [18]. This also makes ASE and ASB more sensitive because other causes of variation between individuals are eliminated [16]. While individually histone QTL (hQTL), expression QTL (eQTL), allele specific expression QTL (aseQTL) and allele specific binding QTL (asbQTL) have been investigated in humans and other animals [19–22], to our knowledge, no studies have systematically investigated and intersected the four molecular QTL in mammals.

We hypothesise that SNPs that affect the height of histone modification ChIP-seq peaks and are associated with the expression of nearby genes are likely to be enriched for causal variants affecting gene expression and perhaps other complex traits. Additionally, as eQTL and other causal variants are enriched under histone modification ChIP-seq peaks [10], we hypothesise that these expression and histone modification QTL will be under peaks. To test this, we identified hQTL, exon expression QTL (eeQTL), asbQTL and aseQTL in mammary tissue from approximately 100 (histone modifications) and 400 (exon expression) dairy cows. The results of these analyses were intersected; first to identify high confidence QTL that were found in two independent analyses, and second to identify SNPs affecting both histone modifications and gene expression - highlighting candidate causal variants. This study identifies variants in the bovine genome which affect gene expression and the height of ChIP-seq peaks and are in the peak whose height they regulate. To confirm these variants are causal we show that they are associated with gene expression in independent data and that they occur in DNA sequences where transcription factors bind.

## Results

RNA-seq data for 411 dairy cows and ChIP-seq data for a subset of 99 of these animals was generated. This data was used to identify traditional QTL (eQTL and hQTL) and allele specific QTL (asbQTL and aseQTL). We intersected these molecular QTL to identify potentially causal variants.

### RNA-seq and ChIP-seq

RNA-seq data for 411 mammary samples is previously described in [23, 24]. For ChIP-seq, there were 99 samples assayed for H3K4Me3, 97 samples assayed for H3K4Me1 and 37 samples assayed for H3K27ac (Supplementary Table 1). All samples had a Jensen Shannon Distance (JSD) more than 0.25, indicating high quality. There were between 36,000 and 940,000 peaks found in each sample. The number of peaks per sample was highly dependent on read depth.

Merged bam files containing between 97 and 199 million mapped reads resulted in a set of consensus peaks for each mark containing between 400 and 700 thousand peaks. JSD values were between 0.4 and 0.5 for these peak sets indicating data were of high quality.

### Allele Specific QTL analysis

For allele specific expression (ASE) and allele specific binding (ASB) analysis, the phenotype was the ratio of maternal to paternal allele counts in the RNA-seq or ChIP-seq data respectively. Maternal and paternal allele counts could only be defined when there were heterozygous sites in the peak (ASB) or exon (ASE). However, there was often more than one heterozygous site in a gene,exon or peak. This resulted in multiple phenotypes for the same feature. To test whether allele counts from multiple SNPs in a peak, gene or exon could be combined we tested the heterogeneity of the maternal:paternal ratio of variants within a peak, gene or exon. Less than 12% of peaks had multiple variants with significantly (*p* < 0.05) different maternal:paternal ratios, 59% of genes and 18% of exons (Supplementary Table 2 and 3). Therefore, although there is some evidence that not all heterozygous sites within the same exon/peak have the same direction of maternal:paternal ratios, the majority did so the allele counts within the same exon/peak were combined and one phenotype was analysed per exon or peak. This was not the case with genes so all allele specific expression analysis was conducted at the exon level.

Although SNPs in the peak (pSNP) or exon (tSNP) are used to define the phenotype, they are not necessarily the causal or driver SNP (dSNP). Therefore, all SNPs within 1 Mb of the peak or exon were tested as a potential dSNP. Linear regression was used to analyse allele specific QTL, however we first filtered phenotypes and dSNPs using a primary test, as the linear regression (the second test) was not sufficient in cases where there were only a small number of animals heterozygous at the dSNP.

In the RNA-seq data, more than 48,000 exons representing 12,716 genes were tested for aseQTL (Table 1) across 337 animals for which there was parental genotypes. 15,308 exons, representing 6569 genes, had at least one significant dSNP (*p* < 0.0001) in both tests. There were almost 2 million aseQTL found.

**Table 1:** Summary of allele specific analysis results including the number of samples assayed, the number of phenotypes tested with the number with at least one significant dSNP (*p* < 0.0001) in the 1st and 2nd tests and the total number of significant dSNPs

	Samples	Number of phenotypes tested	Number of phenotypes with at least one significant dSNP (*p* < 0.0001) 1st Test	Number of phenotypes with at least one significant dSNP (*p* < 0.0001) 2nd Test	Number of significant (*p* < 0.0001) dSNPs
**RNA**	337	48,456	32,565	15,308	1,999,869
**H3K4Me3**	96	224,183	75,261	15,918	1,055,069
**H3K4Me1**	95	220,588	104,627	20,555	981,026
**H3K27ac**	36	283,235	86,487	7963	154,739

In the ChIP-seq data, around 200,000 peaks were tested for asbQTL for each mark (Table 1). Between 7000 and 20,000 of these peaks had at least one significant dSNP (*p* < 0.0001) in both tests. There were up to ~ 1 million asbQTL variants found for each mark.

### Exon expression and histone QTL

For traditional QTL analysis, the phenotype was defined as the number of reads mapping to the peak (hQTL) or exon (eQTL).

Approximately 173 thousand exons (representing 14,504 genes) were tested for eeQTL (Table 2). The increased number tested using this method versus aseQTL analysis reflect the fact that not all exons contained heterozygous variants. More than 66 thousand exons representing 10,696 genes had at least one significant dSNP, resulting in more than 5 million significant eeQTL.

**Table 2:** Summary of traditional QTL analysis results including the number of samples assayed, the number of phenotypes tested and the number with at least one significant dSNP (*p* < 0.0001) as well as the total number of significant dSNP

	Samples	Number of phenotypes tested	Number of phenotypes with at least one significant dSNP (*p* < 0.0001)	Number of significant (*p* < 0.0001) dSNPs
**RNA**	371	173,511	66,275	5,402,049
**H3K4Me3**	96	387,770	23,263	1,234,995
**H3K4Me1**	95	293,902	24,119	1,840,063
**H3K27ac**	36	503,921	11,374	346,175

There were ~ 200–500 thousand peaks tested for hQTL (Table 2). Between 11 and 24 thousand of these peaks had at least one significant dSNP resulting in up to 1.8 million hQTL variants.

### Comparison between allele specific and traditional QTL analysis

The allele specific and traditional QTL (exon expression and histone) test the same phenotypes in different ways. Therefore, we expect overlap between the results, and SNPs significant in both analyses could be considered robust candidates as there were two lines of evidence supporting their association with exon expression or histone modification peak height.

To determine whether the allele specific and traditional QTL analyses were identifying similar effects, peaks with significant asbQTL and hQTL were compared. We observed more overlap between peaks with significant asbQTL and hQTL than expected by chance (Table 3). However only around half the overlapping peaks shared significant dSNPs. When a peak had a dSNP that was significant in both hQTL and asbQTL analyses, the direction of effect was nearly always the same (Table 3).

**Table 3:** The overlap between peaks or exons with significant dSNPs (odds ratio in brackets) and the number of peaks/exons with shared significant dSNPs (*p* < 0.0001) as well as the number of peaks where the direction of the SNP effect was the same in both hQTL and asbQTL analysis. Also the number of exons where the direction of the SNP effect was the same in both eeQTL and aseQTL analysis

	Peaks tested in both	Peaks with significant asbQTL	Peaks with significant hQTL	Overlap	Overlapping peaks with shared significant dSNPs	Peaks with hQTL and asbQTL effects in the same direction
H3K4Me3	224,183	15,918	14,816	3656 (OR = 5.26)	1981	1975
H3K4Me1	220,588	20,555	18,840	4706 (OR = 3.9)	2395	2375
H3K27ac	283,232	7963	5943	243 (OR = 1.5)	59	59
	**Exons tested in both**	**Exons with significant aseQTL**	**Exons with significant eeQTL**	**Overlap**	**Overlapping exons with shared significant dSNPs**	**Exons with eeQTL and aseQTL effects in the same direction**
RNA	48,456	15,308	25,163	10,678 (OR = 2.97)	6733	6676

To determine whether the allele specific and traditional QTL analysis were identifying similar differences in exon expression, exons with significant aseQTL and eeQTL were compared. There was more overlap between exons with significant aseQTL and significant eeQTL than expected by chance (Table 3). A large proportion of these exons had significant dSNPs in the same direction.

### Comparison between gene expression and histone modification QTL

To test our hypothesis that variants associated with the height of ChIP-seq peaks also associate with gene expression, we compared significant asbQTL and aseQTL and significant hQTL and eeQTL.

There was more overlap between asbQTL and aseQTL implicated variants than expected by chance (Odds ratio > 3.1). When all three histone modifications were considered 31% of aseQTL variants were also asbQTL variants for at least one of the marks. In more than half of these cases (more than 57%), the direction of the SNPs effect was the same (Table 4). That is, the allele that increased peak height also tended to increase gene expression.

**Table 4:** The number of SNPs identified in both aseQTL and asbQTL analyses (odds ratio in brackets) and the percentage where the direction of the SNP effect was the same

	Total dSNPs tested	aseQTL	asbQTL	Overlap	Same direction of SNP effect (%)
H3K4Me3	13,397,022	1,999,869	1,055,069	410,022 (OR = 4.3)	61.5
H3K4Me1			981,026	339,821 (OR = 3.4)	57.4
H3K27ac			154,739	53,829 (OR = 3.1)	66

The median distance between an asb/aseQTL and the exon it was associating with was 150Kb. By comparison the median distance between the asb/aseQTL and the peak it was associating with was 50Kb for H3K27ac, 102Kb for H3K4Me3 and 73Kb for H3K4Me1.

By looking at dSNP common to a peak and an exon it is possible to link histone modifications to the genes they are putatively regulating. We found between 5 and 24 thousand peak-exon pairs (representing between ~ 1500 and 3000 genes) for each mark, with a median distance of around 300Kb between the peak and the exon (Supplementary Table 4). There were approximately three peaks per exon and three exons per peak.

There was more overlap between eeQTL and hQTL than expected by chance for H3KMe3 and H3K4Me1 (Odds ratio > 2.4) but not for H3K27ac (Odds ratio = 0.8) (Table 5). However, 29% of eeQTL variants were also hQTL variants for one of the three marks tested. More than 58.9% of the time the direction of effect was the same.

**Table 5:** The number of SNPs identified as both eeQTL and hQTL (odds ratio in brackets) and the percentage where the direction of the SNP effect was the same

	Total dSNPs tested	eeQTL	hQTL	Overlap	Same direction of SNP effect (%)
H3K4Me3	13,397,022	5,402,049	1,234,995	783,358 (OR = 2.8)	58.9
H3K4Me1			1,840,063	1,082,690 (OR = 2.4)	63.3
H3K27ac			346,175	120,207 (OR = 0.8)	59.9

The median distance between an h/eeQTL and the exon it was associating with was 368Kb. By comparison the median distance between the h/eeQTL and the peak it was associating with was 289Kb for H3K27ac, 194Kb for H3K4Me3 and 216Kb for H3K4Me1. We found between 26 and 154 thousand peak-exon pairs for each mark with a median distance of around 600Kb between them (Supplementary Table 5). There were approximately 3 peaks per exon but 10 exons per peak.

### Histone/allele specific binding QTL lie in the peak they regulate

**Fig. 1:**
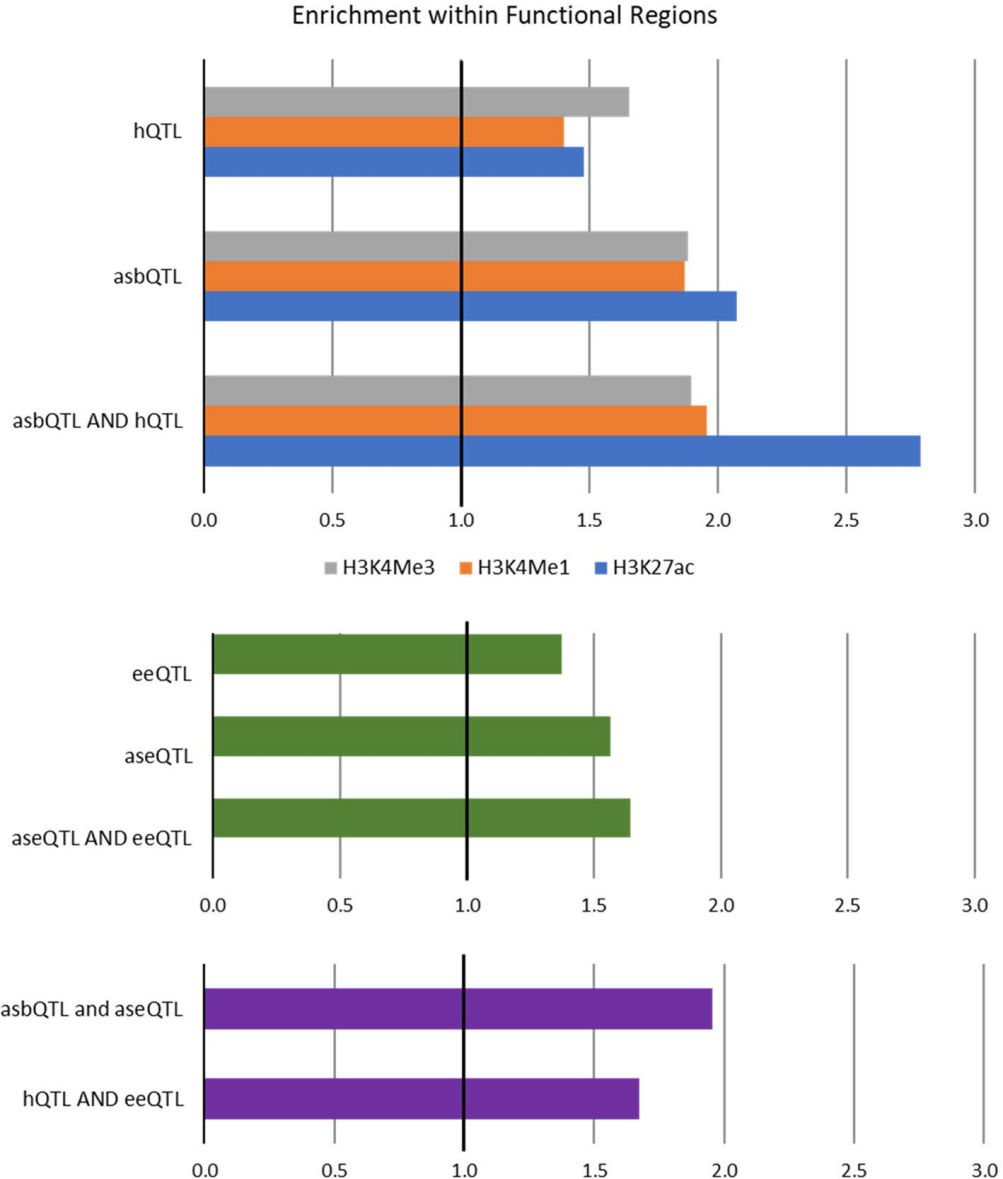
Enrichment (> 1) of significant dSNPs in functional regions of the genome. Top: SNP which were significant hQTL and/or asbQTL for H3K4Me3, H3K4Me1 and H3K27ac were enriched (enrichment > 1) in regions marked by histone modifications. Middle: SNP which were significant eeQTL and/or aseQTL were enriched (enrichment > 1) in regions marked by histone modifications. Bottom: SNP which were significant in both asbQTL (any mark) and aseQTL analysis, or hQTL (any mark) and eeQTL analysis were enriched (enrichment > 1) in regions marked by histone modifications

We hypothesised that causal variants affecting the height of a peak would be found in functional regions of the genome and that these SNPs would most likely be in the peak they were controlling. To test this, we first looked for enrichment of significant dSNPs under any peak. All significant dSNPs were enriched in regions marked by H3K4Me1, H3K4Me3 and H3K27ac (Fig. 1). Allele specific binding QTL were slightly more enriched under peaks than hQTL. Similarly, aseQTL were slightly more enriched under peaks than eeQTL. When considering dSNPs which were both asbQTL and hQTL or aseQTL and eeQTL, enrichment increased considerably in H3K27ac but not in other cases. Significant dSNPs that were both aseQTL and asbQTL or hQTL and eeQTL were also enriched in functional regions.

We also looked to see if significant dSNPs were found under the specific peak they were associated with as this would be consistent with the hypothesis that the variant was altering binding sites in the peak. In up to 20% of peaks with a significant hQTL, the hQTL lay in the peak with which it was associated. (Table 6). In up to 31% of peaks with a significant asbQTL, the asbQTL lay in the peak with which it was associated. When looking at peaks that had a significant dSNP in both analyses, 77% of these peaks had the hQTL/asbQTL in the peak it was associating with (Table 6).

**Table 6:** The number of significant dSNPs (hQTL and asbQTL) which were in the peak they were associating with in each analysis. The number of peaks with the same hQTL/asbQTL in the peak and the number of times a SNP in the peak had the same or lower *p*-value in the asbQTL analysis than the best hQTL

	H3K27ac	H3K4Me3	H3K4Me1
**Peaks with hQTL**	11,374	23,263	24,119
**hQTL variant in the peak**	223 (2%)	2642 (11%)	4770 (20%)
**Peaks with asbQTL**	7963	15,918	20,555
**asbQTL variant in the peak**	490 (6%)	4976 (31%)	4944 (24%)
**Peaks with the same hQTL/asbQTL (in the same direction)**	59	1975	2375
**hQTL/asbQTL variant in the peak**	46 (78%)	1525 (77%)	1830 (77%)
**Peaks with top significant hQTL tested in asbQTL**	2844	10,931	15,150
**PeakSNP better or the same as hQTL in asbQTL analysis**	2029 (71%)	8263 (75%)	12,168 (80%)

If the SNP in the peak was the causal SNP, one might expect it to be significant. However, since there are only one or a few SNPs under each peak, and thousands of dSNPs were tested per peak, random variation in *p*-values may have resulted in distal dSNPs being more significant than a causal SNP by chance. To make an unbiased comparison, the most significant hQTL SNP for a peak was compared to the SNPs under a peak in the asbQTL analysis (Table 6). In the majority of cases the peak SNP was as significant or more significant than the most significant hQTL SNP.

### Putative causal variants

Variants were filtered to create a list of likely causal variants based on the analysis done in this paper. Variants included were significant in the traditional QTL analysis (*p* < 0.0001) and in the first test of the ASB and ASE QTL analysis (*p* < 0.0001). Only variants in the peak they were associating with were included. Lastly the direction of effect of the variants had to be the same in all 4 analyses. This resulted in 12,932 unique SNPs that were associated with histone modifications, *cis* gene expression changes, and were found in a putative functional region. This list was filtered further by combining the *p*-values from each individual test and selecting only the lowest *p*-value for each peak-exon pair, resulting in 4741 unique SNPs (Table 7). These highly curated variants were linked to a total of 1102 genes as differentially regulated in the dataset (Supplementary Table 8). In most cases there were more than one putative causal SNP per histone peak, therefore further filtering may be necessary but this was not performed in this study.

**Table 7:** Candidate causal variants based on the analysis done in this paper. The number of peaks and genes they are associated with. There were 4741 unique candidate causal variants identified, however a small number of these were associated with more than one mark

	Number of putative causal variants	Number of peaks	Average putative causal SNPs identified per peak (Average size of peak)	Number of exons
H3K27ac	146	47	3.1 (2004)	173
H3K4Me3	1675	633	2.7 (1460)	1720
H3K4Me1	3158	874	3.6 (4073)	1917

**Fig. 2:**
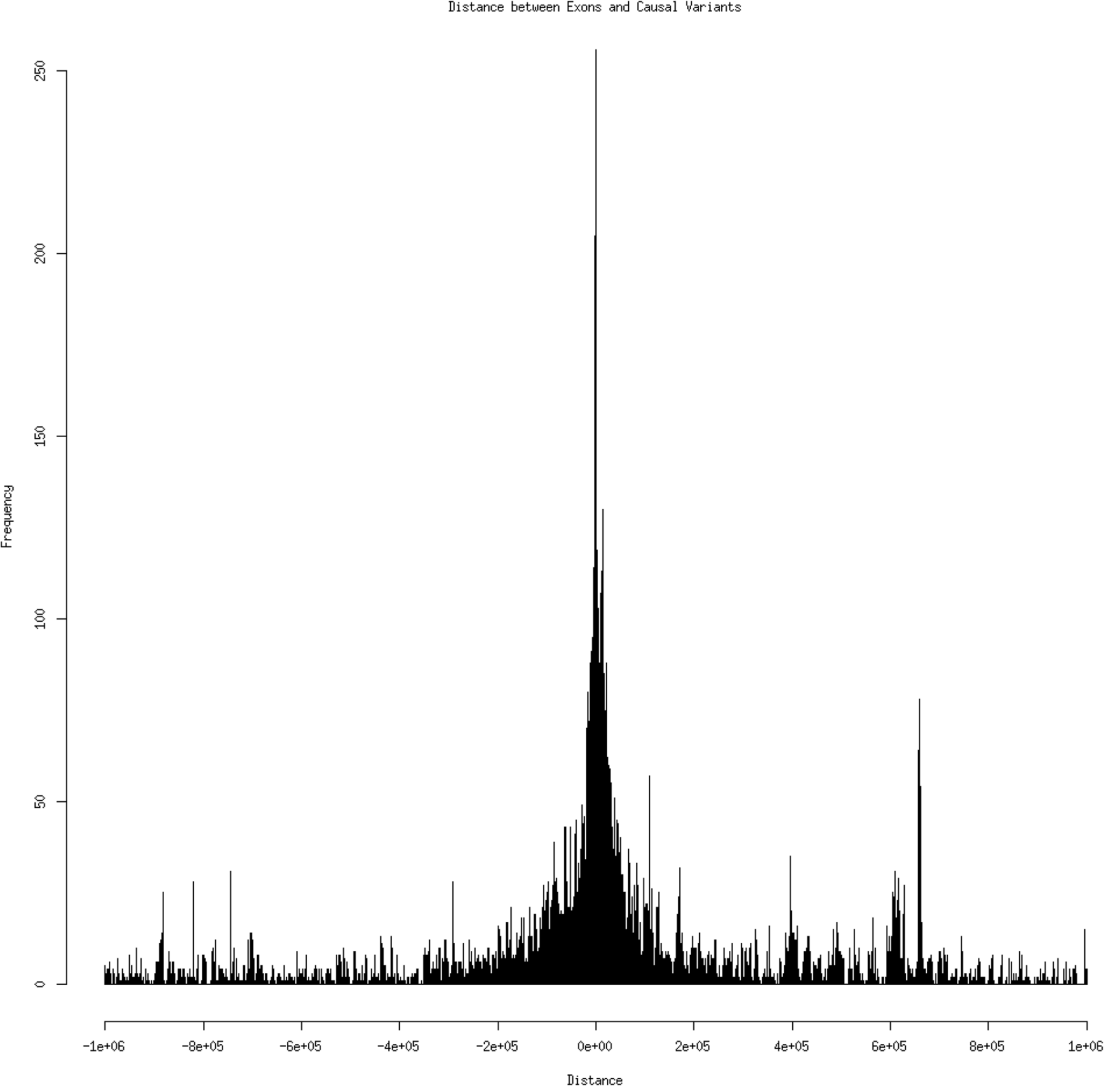
Histogram of the distance between the candidate causal variants identified in this paper and the exon they are associated with (as both eeQTL and aseQTL)

Most candidate causal SNPs (50%) were less than 50Kb from the exon they were affecting (Fig. 2), however there were still putative causal SNPs up to 1 million base pairs away.

The 12,932 unique SNPs that were associated with histone modifications, *cis* gene expression changes, and were found in a putative functional region as described above were compared to results from the cattle Genotype-Tissue Expression (cGTEx, [25]) data for gene expression in the mammary gland and in blood. Not all the variants and genes tested in our data were included in the cGTEx data (Table 8). However, there was very high agreement in the direction of effect for variants that overlapped between the two datasets.

**Table 8:** Proportion of putative causal SNPs also reported as expression QTL in the cattle GTEx data in mammary tissue and blood cells

	Putative causal variants	cGTEx tissue type	Variant and gene tested in cGTEx	cGTEx *p* < 0.01	Same direction of effect at *p* < 0.01
H3K27ac	391	Mammary	269	149	149
		Blood	327	115	111
H3K4Me3	3554	Mammary	2219	707	691
		Blood	2008	725	578
H3K4Me1	9835	Mammary	6022	1254	1201
		Blood	5405	1534	1306

### Identification of putative binding motifs

The sequence under aseQTL and asbQTL was analysed to identify putative transcription factor binding motifs. Starting with 101,726 significant aseQTL and asbQTL variants representing 897 exons and 3143 ChIP-seq peaks, a total of 2553 groups of clustered sequences were identified (comprising ≥10 sequences each), with 1165 generated from aseQTL and the remainder from asbQTL (174, 759, and 455 for H3K27ac, H3K4Me1, and H3K4Me3 respectively). These represent common sequence motifs underlying variants associated with differences in gene expression and peak height.

**Fig. 3:**
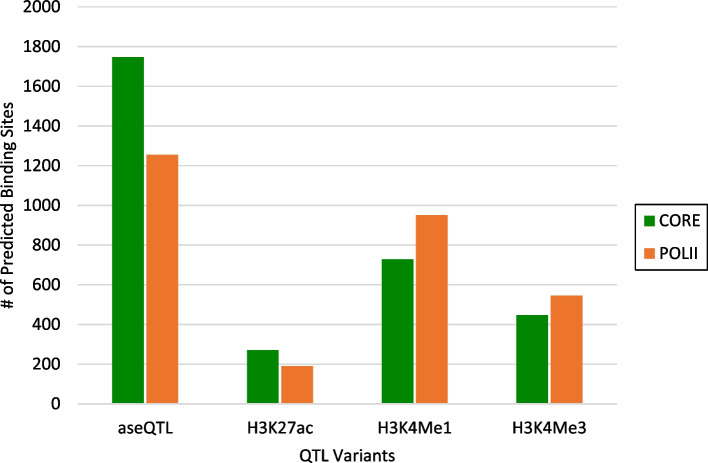
The numbers of predicted TF binding sites by JASPAR 2018 database (CORE or POLII) for motifs identified by clustering significant aseQTL or asbQTL variants

To assess whether the motifs identified represent real functional biology, we attempted to identify transcription factors with binding sites matching the motif sequences. Across these groups, a total of 6134 putative TF binding sites were identified (Fig. 3), with a total of 2069 groups (81.0%) producing at least one predicted TF binding site. For both the aseQTL and H3K27ac clusters, the majority of predicted binding sites were from the CORE database of transcription factor binding sites. For the two methylation phenotypes H3K4Me1 and H3K4Me3, the majority of predicted sites were from the POLII database of core promoter elements.

Considering only the predicted binding site for each group that had the highest relScore, the largest proportion of CORE elements identified belonged to the *C2H2* zinc finger family (ten most frequent families listed in Supplementary Table 6), with the most frequently identified TFs from this family being MZF1, ZNF354C, ZNF384, HIC2, and ZNF740. A total of 31 transcription factors from the Krüppel-related zinc finger family were also predicted. Among the Homeo domain factors, the most commonly predicted families were NK-related and HD-LIM factors (26 and 14 respectively out of 76 total), and the Rel homology factors were almost completely accounted for by the NFATC1 and NFATC2 transcription factors (28 and 10 respectively, out of 43).

Supplementary Table 7 shows the most-frequently detected core promotor elements (From the POLII database), again considering only the predicted TF with the highest relScore for each group. The most common element was the downstream B recognition element (BREd).

When considering only the putative causal variants that were significant under all four QTL analyses (eeQTL, aseQTL, hQTL, and asbQTL), the majority of predicted binding sites for both the H3K4Me1 and H3K4Me3 variants were predominantly from the CORE database (54.9 and 65.9% respectively), in contrast to the results presented above for all significant asbQTL variants. No clusters with more than five sequences could be identified for the H3K27ac variants, so no motifs or TF binding sites were generated for these variants.

To investigate whether the clusters identified represented real biology, two further analyses on the motif sequences were conducted. First, to test for skew in the composition of bases at the central position of the motif (i.e., the location of the variants of interest), a multinomial test of reference and positive allele frequencies was conducted. Bases representing the binding or expression increasing allele were selected for each sequence that made up the motif. Base compositions at this location which were skewed towards one or two bases, and therefore showed higher significance against a null hypothesis of uniform composition, were theorised to represent motifs where changes would affect binding or expression. Table 9 shows the percentage of groups where the positive effect alleles were significantly skewed (LLR *p*-value < 0.05), and a comparison to the same statistic calculated using the allele from the reference genome for each sequence: the reference alleles were assumed to be relatively random, as they are based on the genotypes of the individual animal that was chosen as the reference. The higher percentage of groups showing significant skew observed in the positive effect alleles suggests that the positive-effect allele is more likely to be conserved and therefore more likely to be biologically important. However, these alleles are not conserved across motifs: for example, some motifs had positive effect alleles heavily biased towards A and T, while others had equally heavy bias towards G or C.

**Table 9:** The percentage of motif clusters where the base composition was significantly (LLR *p*-value< 0.05) biased towards the positive and reference alleles

	aseQTL	H3K27ac	H3K4Me1	H3K4Me3
Positive Allele	57.9%	57.5%	56.5%	56.3%
Reference Allele	12.2%	11.5%	5.5%	10.5%

The second analysis of the motif sequences looked for enrichment of the motif consensus sequences relative to their expected frequencies in the bovine genome, on the basis that sequences representing TFBS may be over-represented compared to random non-functional sequences of the same length and base composition. For each motif, a consensus sequence containing IUPAC ambiguity codes was generated, then converted into a regular expression that was subsequently matched against the reference genome, and the number of matches used to represent the observed genomic abundance of the motif. The expected abundance was calculated assuming base frequencies of 0.291 for A and T, and 0.209 for C and G, as observed for the autosomes of the ARS-UCD1.2 bovine reference. This analysis showed a median enrichment of 2.3× (geometric mean: 3.6×) for observed motif sequences in the genome. When restricted to sequences within 10Kb of a TSS, higher levels of enrichment were observed (median 2.8×, geometric mean 4.2×), although this extra enrichment disappeared (median 2.3×; geometric mean 3.5×) when the expected counts were calculated using base frequencies from the same regions (0.272 for A and T; 0.228 for C and G). Overall, these results suggest that the motif sequences are over-represented in the genome, particularly near TSS.

### Molecular QTL modulate key lactation genes and phenotypes

Previous GWAS of lactation traits have highlighted a number of large effect QTL in cattle, implicating genes and loci that underpin substantial variation in the yield and composition of milk. It is therefore noteworthy that many familiar candidates were highlighted in our analysis of putative causal variants above (Supplementary Table 8), with genetic co-regulation of expression and histone modification suggested for these key lactation genes. A non-exhaustive list of these candidates includes *ABCG2* [26], *ABO* [27, 28], *ANKH* [24, 29], *BTN1A1* [30], *CSF2RB* [31, 32], *GHDC* [24], *KCNJ2* [24], *LRRC8C* [24], *LTF* [33], *PAEP* [34], *PICALM* [24, 29], *SLC37A1* [35], *STAT5B* [32], and *XDH* [28], and relaxing our relatively stringent criteria that candidates should present significant molecular QTL for all 4 association tests might be expected to identify further such genes.

**Fig. 4:**
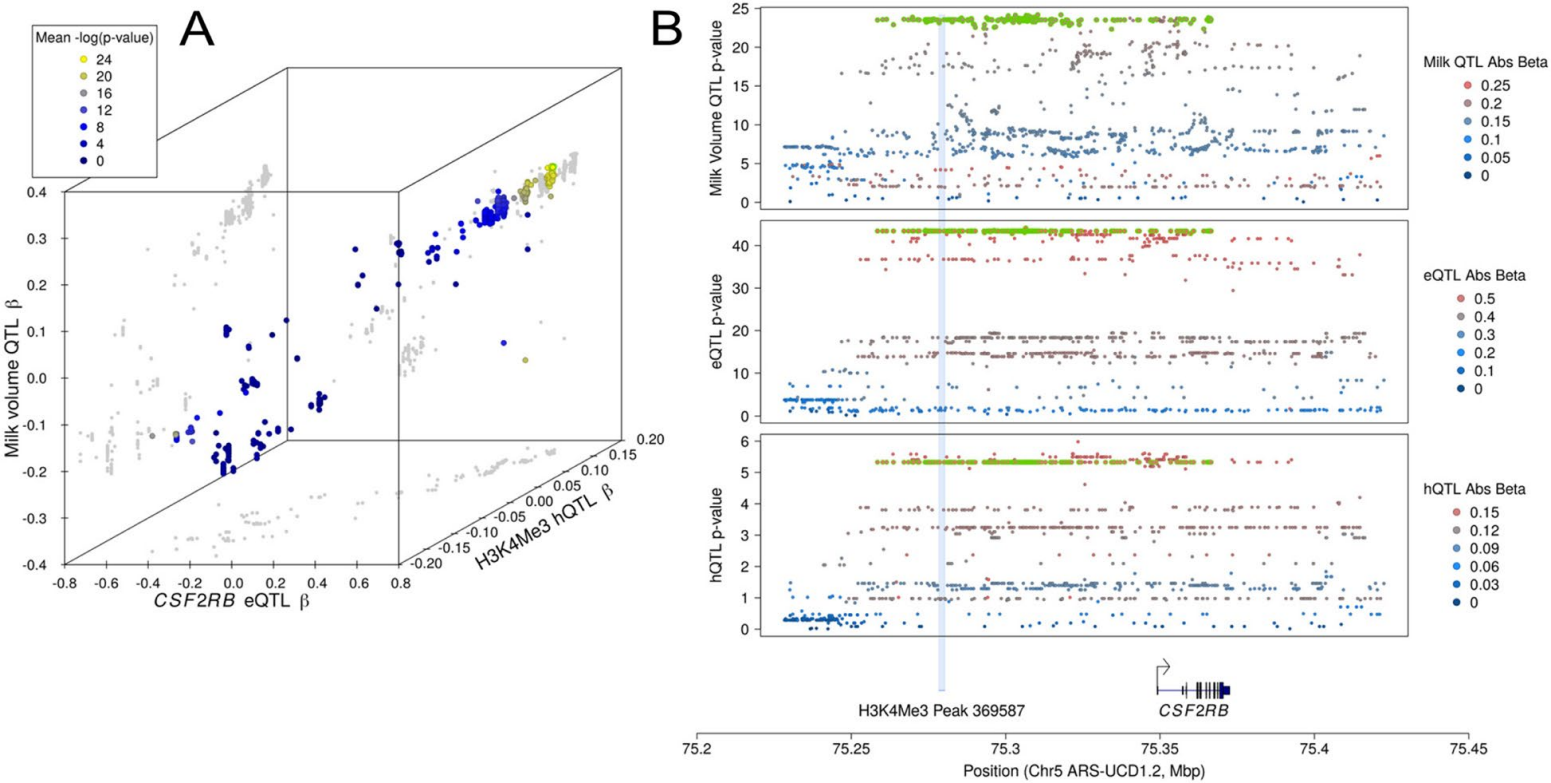
A) Three-dimensional scatter plot showing effect estimates for milk volume, CSF2RB gene expression and H3K4Me3 peak (chr5:75,278,382–75,280,245) height for 1446 sequence variants. Variant *P*-values of association are indicated by colour. Variants that were in the top 80% (ranked by beta) for all three QTL are circled in green. B) The same data visualised by Manhattan plots, showing location details of associated variants, boundaries of the H3K4Me3 mark, and the CSF2RB gene. In these plots, *P*-value is represented on the Y-axis, with colour indicating variant beta-estimates. Variants that were in the top 80% (ranked by beta) for all three QTL are circled in green

The utility of histone modification data for fine-mapping candidate causative variants for genes important to lactation is particularly apparent at the *CSF2RB* locus. The *CSF2RB* gene has been previously shown to regulate milk volume yield and the fat and protein percentage of milk [24, 32]. Analysis of this hyper-variable locus presented a haplotype comprising a very large number of tightly linked variants as candidates (*N* = 365 at R2 > 0.9 across all traits), making further prioritisation challenging [31]. The expression-based mechanism of the QTL is apparent due to the co-location of a strongly correlated eQTL [24], where in the current analysis, we similarly observe QTL for histone modifications (Supplementary Table 8). To assess the potential inter-relatedness of these effects, we re-analysed milk yield QTL data from the analysis reported by Lopdell, Tiplady [31] (*N* = 29,350 cows) (Fig. 4). This analysis highlighted an H3K4Me1 mark at chr5:75,302,529–75,305,459, and H3K4Me3 mark at chr5:75,278,382–75,280,245 as potentially responsible for these effects, showing significant hQTL/asbQTL that respectively encompass 21 and four of the previously highlighted milk yield-associated variants (the location and hQTL of the H3K4Me3 peak is shown in Fig. 4b). The association statistics of these 25 variants also places them at the top and near-top of the hQTL/asbQTL, presenting a subset of variants that are strong candidates for the molecular and lactation QTL ascribed to the locus.

## Discussion

It is difficult to identify causal variants for complex traits, including gene expression, because there is widespread LD between the causal variants and other variants [36]. Evidence of causality that was not affected by LD would help the search for causal variants greatly [3]. Putative regulatory regions of the genome, identified by ChIP-seq, are known to be enriched for causal variants and are independent of LD and so provide information on which variants to prioritise as potentially causal [37, 38]. However, functional regions cover a large fraction of the genome and so still leave many polymorphisms as possible causal variants [11]. The list of possible causal variants can be further reduced by focussing on variants which affect the height of the ChIP-seq peaks as this indicates these SNPs are affecting binding at potential regulatory regions which may be controlling gene expression [17]. Here we examine the hypothesis that variants under ChIP-seq peaks that affect peak height are enriched for causal variants affecting gene expression. To do this we used both conventional association analysis to find hQTL and eeQTL, and analysis of allele specific expression of genes (ASE) and allele specific binding of modified histones (ASB) measured as differences in ChIP-seq peak height. For this analysis 233 ChIP-seq samples were generated from mammary biopsies from around 100 dairy cows. All were assayed for H3K4Me3 and H3K4Me1, and a subset of approximately 30 animals, for H3K27ac. RNA-seq data for 411 samples (99 of which were assayed with ChIP-seq) was also obtained. To our knowledge this is the one of the largest studies to describe hQTL in tissues and the first time hQTL or asbQTL have been identified in dairy cows.

Allele specific analyses complement ‘traditional’ eQTL and hQTL analyses because they test the same phenomena (*cis* eQTL and hQTL) using independent methods [18]. However, there can be real differences between allele specific and traditional analyses. For instance, allele specific analyses can detect parent of origin effects (imprinting) and traditional analyses can find *trans*-acting QTL [16, 39]. In our study, only a small proportion of peaks with significant dSNPs had the same dSNP as an asbQTL and hQTL (Table 3). A slightly larger proportion of exons with significant dSNPs had the same dSNP as an aseQTL and eeQTL. We believe these differences are largely due to lack of power as the overlap is greater in the larger, more powerful gene expression dataset (*n* = 371). In H3K4Me1 and H3K4Me3, more than 50% of the peaks with significant dSNPs in both analyses found the same SNP as a hQTL and asbQTL (Table 3). This was lower in H3K27ac (24%) possibly due to a smaller sample size restricting which SNPs met significance thresholds. In all 3 marks, 99–100% of shared asbQTL and hQTL had the same direction of SNP effect. In the gene expression analysis, there was also an enrichment of exons with significant dSNPs in both aseQTL and eeQTL analysis (Table 3). More than 60% of these exons had shared significant dSNPs and 99% of the time the direction of effect by the aseQTL and eeQTL were the same. These SNPs, identified as aseQTL and eeQTL for the same exon or asbQTL and hQTL for the same peak, have been found in two independent analyses so are excellent candidate regulatory variants.

We hypothesised that SNPs that affect ChIP-seq peak height are also likely to affect gene expression as the regions marked by the histone modifications may be regulating gene expression [12]. In this study, the overlap between asbQTL and aseQTL, as well as hQTL and eeQTL was more than expected by chance (Table 4). Up to a third of genetic variants associated with differences in gene expression were also associated with differences in histone modification binding in one or more of the three marks tested. Although highly dependent on sample size this proportion is similar to that seen in other studies [40], but lower than some others [17]. This supports the hypothesis that the same causal variants affect peak height and gene expression. More than 57% of the time the direction of effect of the shared variant was the same, for example an allele that increased peak height also increased exon expression (Table 4). This suggests the role of these histone modifications is primarily activating, which is consistent with the hypothesised role of these marks [40]. We connected peaks with exons that shared a QTL. Consistent with indications that functional regions such as enhancers can work over long distances [17, 41] 50% of peak-exon pairs were more than 400Kb away from each other (Supplementary Tables 4 and 5). We found that peaks were regulating multiple exons which is not surprising as these may be from the same gene, or regulatory regions could be affecting multiple genes (as described in Pott and Lieb [42]). Interestingly, some exons appeared to be regulated by multiple peaks. This is compatible with evidence that one gene can be regulated by multiple functional regions [43]. By intersecting the results of QTL for gene expression and histone modifications, we identified putative causal variants which altered gene expression and affected histone modification of putative regulatory regions in the bovine genome. Additionally, by comparing QTL affecting histone modification with QTL affecting exon expression we link regulatory regions to the exons they are regulating.

In order to compare traditional QTL for a feature such as a peak where there is one phenotype, to allele specific QTL for the same feature, which has multiple phenotypes at multiple SNP under the feature, it was necessary to combine allele counts under each feature for the allele specific analysis. We found that there was less variation between allele counts in exons than in genes (Supplementary Table 2), therefore we conducted our analysis (for both traditional and allele specific analysis) at the exon level. In doing so there is a danger that some variation in exon expression is because of variation in splicing which would not necessarily be associated with histone modification levels. However, other studies have found that many exon expression QTL are also gene expression QTL (Xiang et al. 2018, Guan et al. 2014) and we observed more overlap between QTL associating with exon expression and QTL associating with histone modification peaks than expected by chance.

Identification of causal variants is still difficult because both histone and expression QTL analyses are affected by the same LD, so we need evidence of causality that is not affected by LD. However, if the causal variants are located in the peak whose height they regulate, this would be evidence independent of LD. Numerous studies have shown that putative causal variants for a variety of complex traits are enriched in functional regions of the genome [9, 37, 44–47]. Consistent with this, we found that all four QTL results were enriched in ChIP-seq peaks for H3K27ac, H3K4Me1 and H3K4Me3 (Fig. 1). Similarly, SNPs which were linked to gene expression and histone modification binding (either asbQTL and aseQTL or hQTL and eeQTL) were also enriched under peaks. However, we hypothesised that causal variants for peak height were likely to be found in the peaks they were associated with as they may be altering regulatory binding sites in these locations. In H3K4Me1 and H3K4Me3 there were a large proportion of significant dSNPs found in the peak they were associated with in both hQTL (12–20%) and asbQTL (24–31%) analyses (Table 6). However, in some cases significant dSNPs were found up to 1 Mb away from the peak they were associated with. As large numbers of SNPs were tested it’s possible the small number of SNPs in the peak could not compete with the large number of SNPs tested outside the peak. We examined this hypothesis by comparing the most significant SNP from the hQTL analysis with a SNP in the peak in the ASB analysis. In this one-to-one comparison, the SNP in the peak almost always (71–80%) had a better than or equal *p*-value than the most significant SNP from the hQTL analysis for all 3 histone modifications (Table 6). Similarly, when considering the SNPs that were significant in both hQTL and asbQTL analysis for which there was high confidence, 77% of peaks had a significant asbQTL/hQTL in the peak. This provides evidence that dSNPs under peaks were affecting peak height. This work confirms that gene expression QTL are enriched under peaks and finds that hQTL and asbQTL were often found in the peak whose height they affected. Therefore, when looking for causal variants it would be sensible to filter for variants under the ChIP-seq peak.

If ChIP-seq peaks contain binding sites for transcription factors and if mutations in these sites cause hQTL/asbQTL and/or eQTL/aseQTL, then the QTL discovered may share the same DNA sequence because they are bound by the same transcription factor [17]. In addition, if two sites, heterozygous for the same allele, under different peaks affect binding of the same transcription factor, then the same allele should be associated with the higher ChIP-seq peak in both cases. This is what we found, asbQTL and aseQTL sites share common sequence motifs, which are enriched in the genome and (for asbQTL) the allele associated with the higher peak is partially conserved across peaks sharing a similar motif (Table 9). A large proportion of these motifs also correspond to predicted TFBS consensus sequences, with a large subset of these belonging to the Krüppel-like family (KLF) of transcription factors. Several of these TFs, including KLF9 (predicted to bind 10 motif clusters), KLF13 [2], KLF14 [2], and KLF16 [9] contain binding domains for the regulatory protein Sin3a, which in turn has been shown to interact with a range of histone deacetylase [48, 49] and chromatin remodelling [48, 50] enzymes, providing a clear potential mechanism by which these sites could influence rates of gene expression and histone modification. Another twelve motifs match predicted binding sites for the Basic helix-loop-helix factor (bHLH) Max, which similarly interacts with Sin3a and Sin3b to promote histone deacetylation [49].

We conclude that exon expression QTL are often also histone modification QTL and that histone modification QTL are often in the peak they regulate. Based on these findings we identified a list of SNPs which we argue are likely causal variants for gene expression in the mammary gland (Table 7). These SNPs were affecting peak height and exon expression, suggesting they are affecting gene expression by altering histone modification binding. Although it’s not implausible a SNP that decreases histone modification binding would increase gene expression and vice versa we assumed this would be the minority case, so we only included SNPs that had the same direction of effect on the peak and exon expression. Only SNPs that were found in both traditional and allele-specific analysis were included. As these studies were independent [18] this means there were four lines of evidence pointing to these SNPs as causal variants. Lastly to filter this list further only SNPs which were in the peak they were affecting were included. We compared these results to independently identified gene expression QTL [25] in mammary (from 175 samples) and found between 20 and 55% of gene-SNP pairs were significant and more than 85% of the time the direction of effect was the same (Table 8).

While we have demonstrated clear co-occurrence and enrichment of genetic signals for ChIP-seq and gene expression QTL, the question remains what role these effects may have on physiological phenotypes. We did not attempt to undertake a systematic analysis in this regard but note that many genes with prior implication in lactation phenotypes were highlighted in the current study (Supplementary Table 8). The *CSF2RB* gene in particular was of interest, representing a highly variable region with a large number of near perfectly linked, highly associated candidate variants for milk yield and other phenotypes. Using previously published milk yield data from the Lopdell, Tiplady [31] study, we observe the potential mechanistic basis of these lactation impacts, where the size and sign of variant effects suggest coordinated regulation of histone status, gene expression, and consequently - differences in milk yield (Fig. 4). Reducing the number of plausible candidates from hundreds down to tens of variants demonstrates the potential utility of histone QTL data, though we acknowledge that these prioritisation criteria necessarily assume that causal variants locate in the peaks that they regulate. While large-scale functional screens suggest that this will often be the case, regulation in *trans*, and temporal and cell-specific expression of QTL effects means that no single method is likely to capture all possible candidates. Additional layers of omic data and lab-based functional testing are thus anticipated to improve candidate identification further and give the most comprehensive view of the variants regulating complex traits.

## Conclusions

Identifying causal variants from eQTL studies is challenging because it is difficult to filter causal variants from those in LD with them. We propose that variants associated with gene expression which are also associated with differences in histone modification binding are good candidate causal variants for regulation of gene expression in dairy cows. After undertaking hQTL, eeQTL, asbQTL and aseQTL analysis, we found that allele specific and traditional QTL analyses largely target the same phenomena and so combining data from both increases power. We also found that variants putatively causing differences in ChIP-seq peak height are often located in the peaks whose height they affect and that these variants (and eQTL) are often part of DNA sequences we identified as TFBS. Significantly, we found that gene expression QTL were enriched as histone modification QTL, thus providing evidence that non-coding functional regions regulate gene expression. Lastly, we intersected the results of the four independent QTL analyses to identify strong bovine candidate causal variants for gene expression in the mammary gland. This work highlights a novel way to identify causal variants affecting gene expression and potentially other complex traits and adds to the growing research that functional non-coding regions of the genome contain causal variants.

## Methods

### Variant Calling and Genotyping

Whole-genome sequencing was performed as previously described [51] for 1300 animals, to form a reference population for sequence imputation. Briefly, animals with a mixture of Holstein-Friesian (HF; *N* = 306), Jersey (J; *N* = 219), HF × J (*N* = 717), or other breeds and cross breeds (*N* = 58) were sequenced on Illumina HiSeq 2000 instruments targeting 100 bp paired-end reads. Genome sequence data were aligned to the ARS-UCD1.2 genome assembly [52] using BWA-MEM (version 0.7.17, [53]). Variant calling was conducted using GATK HaplotypeCaller (version 4.0.6.0, [54]) with variant quality score recalibration applied. After filtering, this variant set (21,005,869 variants) was phased using Beagle (version 5.0, [55]) to create an imputation reference panel.

A separate, non-overlapping population of 411 animals was used for RNA sequencing (RNA-seq) of which 99 were also used for Chromatin Immunoprecipitation followed by sequencing (ChIP-seq). The majority of these animals had previously been genotyped using the Illumina BovineHD SNP-chip. The remaining subset of 27 cows had been genotyped on a lower density panel (Illumina Bovine SNP50 BeadChip). Imputation to WGS resolution was performed as part of a larger study [51] using the same reference population as described above, and resulted in a variant set of 16,640,294 variants following post-imputation filtering to remove variants with minor allele frequencies less than 0.01 in the 99 animal subset.

For the allele specific analysis, SNP alleles were phased as maternal or paternal. Homozygous genotypes remained unchanged but for heterozygous genotypes, alleles were defined as maternal or paternal based on the sire genotypes. If the sire was homozygous for an allele, that allele was designated as paternal, if the sire was heterozygous the allele was defined based on the phasing with the previous SNP.

### Masked Genome

To prevent bias to the reference alleles when mapping reads to the reference genome, a masked genome was created with a neutral allele for SNPs that were heterozygous in the sequenced animals. Imputed genetic variants described above were filtered for 1% minor allele frequency in this sample set. Data for the 99 ChIP-seq animals were then extracted and filtered again at 1% minor allele frequency. This set of variants (*N* = 14,536,882) was used to create a masked genome where a non-variant allele was placed at that location in the reference genome of ARS-UCD1.2 [52].

### ChIP-seq and RNA-seq

Mammary biopsies and RNA sequencing were performed as reported previously [23, 24]. Briefly, high-depth mammary RNA-seq was conducted on tissue from 411 cows, sampled in three batches at different points in time. Following library preparation, samples were sequenced using the Illumina HiSeq 2000 instrument to produce 100 bp paired-end reads, multiplexed at two samples per lane.

Prior to mapping, reads were processed using Trimmomatic (version 0.39, [56]) in paired-end mode, with settings LEADING:20 TRAILING:20 SLIDINGWINDOW:3:15 MINLEN:50. Processed reads were mapped against the masked genome described above using STAR (version 2.7.0, [57]) in two stages. In the first stage, exon and junction information from the RefSeq database (annotation release 106 [52]) of protein-coding genes was used to produce an initial mapping, which in turn was used to identify additional novel exons and splice junctions for remapping in the second stage. This resulted in a median of 39 million uniquely mapped read-pairs per cow.

ChIP-seq was performed on a subset of 99 animals from the 411 RNA-seq animals, utilising duplicate biopsies obtained at the same time as samples used for RNA extraction and gene expression analyses. Whole frozen tissue samples (weighing between 6 and 37 mg) were fixed for 10 minutes with 10% formaldehyde and chromatin prepared using the Magnify Chromatin Immunoprecipitation kit (ThermoFisher) as per the manufacturer’s instructions. Fixed chromatin was sheared to 200-500 bp using the Covaris S2 (Covaris) for 3 min, duty cycle five, % intensity four and 200 cycles per burst.

Chromatin immunoprecipitation was performed using the Magnify Chromatin immunoprecipitation kit (ThermoFisher) with some modifications. 30ul of sheared chromatin was immunoprecipitated with 0.25μg of antibody. Depending on the amount of sample each reaction was performed 1,2 or 3 times and the samples were combined after de crosslinking using the MinElute PCR purification kit (QIAGEN). Sequencing libraries were prepared for each ChIP sample and a control for each chromatin preparation (input sample) using the NEBNext Ultra II DNA Library Prep Kit for Illumina (New England Biolabs) as per the manufacturer’s instructions and run on the HiSeq 3000 (Illumina) in a 150-cycle paired end run.

Each library was sequenced to between 20 and 200 million reads (median 58 million). Raw sequence reads were trimmed of adapters and poor-quality ends using Trimmomatic (version 0.38, [56]). Bases of quality less than 20 were removed from the 3′ and 5′ ends of the sequence and trimmed reads with length less than 50 were removed. Trimmed reads were mapped to the masked ARS-UCD1.2 genome [52] using BWA-MEM (version 0.7.17-r1188, [53]) with default settings. Poor-quality reads with q < 15 were removed using Samtools (version 1.9, [58]), and duplicate reads were also removed. MACS2 (version 2.1.1, [59]) with default settings was used to call peaks from mapped ChIP-seq reads with input reads as control. The quality of peaks was checked with deepTools plotFingerprint (version 2.5.4, [60]) and SPP (version 1.0, [61]).

To generate a consensus set of peaks for each mark across all samples, equal numbers of reads from each bam file were randomly sampled and merged using Samtools (version 1.9, [58]). Peaks were called from the merged bam file using MACS2 (version 2.1.1, [59]) with default settings as described above.

### Allele Counts

For the allele specific analysis, maternal and paternal read counts were calculated by counting maternal/paternal alleles for all SNPs under a ChIP peak (peak SNP or pSNP) or within an exon (transcript SNP or tSNP) from mapped ChIP/RNA-seq reads using GATK tools (version 4.1.2, [54]. First, a gVCF file was created at base pair resolution using GATK HaplotypeCaller. Then, allele counts were calculated at each SNP under a peak or in an exon using GenotypeGVCF, applying the option “depth per allele by sample”. Individuals genotyped as homozygous at the pSNP/tSNP were excluded from the analysis because they were not informative for allele-specific analyses. Individuals genotyped as heterozygous in the genomic data but were monoallelic at the pSNP/tSNP in the RNA/ChIP data were also excluded to remove potential imputation errors.

To test whether these allele count phenotypes from the same peak/exon shared paternal or maternal allelic bias, all phenotypes under a peak/exon were tested for similarity using a G-test.

For s pSNPs under a peak (or tSNPs in an exon):

Let

n_ij_ = allele count for pSNP i where i = 1 to s and j = maternal or paternal.

n_i._ = total number of counts for SNP i.

n_.j_ = total number of maternal or paternal alleles over all s SNPs.

n_.._ = total of all counts.

These make an sX2 contingency table. To test the null hypothesis that the ratio of maternal to paternal alleles is the same for s SNPs.

*G* = 2( ∑ [*n*_*ij*_ ∙ ln(*n*_*ij*)_] +  ∑ [*n*_.._ ∙ ln(*n*_.._)] −  ∑ [*n*_.*j*_ ∙ ln(*n*_.*j*_)] −  ∑ [*n*_*i*._ ∙ ln(*n*_*i*._)]).

Read counts for peaks/exons with multiple p/tSNPs were calculated as the sum of maternal and paternal allele counts for all p/tSNP in the peak/exon.

### Allele Specific QTL analysis

All SNPs within one megabase (1 Mb) of the midpoint of the peak/exon were tested for association with the phenotype (maternal and paternal allele counts at the exon or peak). We called these SNPs the driver SNPs or dSNPs. Two statistical tests were used to assess the significance of the relationship. The first test was used to filter for cases where sample size was small.

For the first test, where:

0 = reference allele and 1 = alternate allele.

For each individual i that had dSNP genotype 1|0, let M_i_ = number of counts of the maternal allele at the phenotype.

For each individual i that had dSNP genotype 1|0, let P_i_ = number of counts of the paternal allele at the phenotype.

For each individual j that had dSNP genotype 0|1, let N_j_ = number of counts of the maternal allele at the phenotype.

For each individual j that had dSNP genotype 0|1, let Q_j_ = number of counts of the paternal allele at the phenotype.

And *T* =  ∑ *M*_*i*_ +  ∑ *P*_*i*_ +  ∑ *N*_*j*_ +  ∑ *Q*_*j*_ and *A* =  ∑ *M*_*i*_ +  ∑ *Q*_*j*._

Then:

$Z=\left(\frac{A}{T}-0.5\right)\times \sqrt{4T}$ would be approximately normally distributed.

*P* values for the normal distribution were calculated in R (version 3.6.1, [62]). dSNPs which were significant at *p* < 0.001 were taken through to the second test.

For the second test:

We calculated a linear model: *Y*_*i*_ = *a* + *bX*_*i*_

Where, for each individual i,

$${Y}_i= lnln\ \left(\frac{maternal\ allele\ count+10}{paternal\ allele\ count+10}\right)$$

When individual i had dSNP genotype 0|0 or 1|1

$$Let\ {X}_i=0$$

When individual i had dSNP genotype 1|0

$$Let\ {X}_i=1$$

When individual i had dSNP genotype 0|1

$$Let\ {X}_i=-1$$

*P* values were calculated in R [62].

### Histone QTL

Read counts for both the ChIP and Input BAM files were counted for each of the corresponding ChIP-seq consensus peaks. As an initial quality control filter, peaks were removed where the peak read count was below the 1% quantile across all peaks. To remove peaks that were potentially caused by artefacts in the reference genome, additional peaks were removed where the input read depth was more than five times the average across all peaks. This yielded a data set comprising peak and input read counts for 503,921, 293,903, and 387,770 peaks (for H3K27ac, H3K4Me1, and H3K4Me3 respectively).

Next, phenotypes suitable for mixed linear model analyses were generated. First, each Peak (*P*_*ij*_) and Input (*I*_*ij*_) read count was normalised by dividing by the mean read count per animal (across all peaks) to yield normalised counts (*PN*_*ij*_ and *IN*_*ij*_) across all peaks *i* and animals *j*. Ordinary least squares (OLS) was then applied to remove the effect of the Input read depth, in the following manner: for each peak *i*, let *y*_*i*_ = *lnln* (*PN*_*i*, ∙_ + 1) and *x*_*i*_ = *lnln* (*IN*_*i*, ∙_ + 1) , then fit the model *y*_*i*_ = *α* + *βx*_*i*_ + *ε*_*i*, ∙_. The vector of residuals *ε*_*i*, ∙_ was then used as the phenotype for histone QTL (hQTL) discovery.

Prior to association analysis, further filtering was applied to remove outlier samples. Here, individuals were removed using principal components analysis (PCA) criteria, in an approach similar to that employed by Ellis, Gupta [63], those animals with PCA values more than four standard deviations from the mean in any of the first seven components were excluded. This filter yielded a data set containing 34, 96, and 97 cows for H3K27ac, H3K4Me1, and H3K4Me3 respectively. Of these, 33, 94, and 95 respectively had imputed sequence-resolution genotypes available for subsequent analyses.

Histone QTL discovery was performed using GCTA (version 1.93, [64]), applying mixed linear model association testing using the ‘leave one chromosome out’ approach to avoid double fitting of variants of interest (MLMA-LOCO). The genomic relationship matrix (GRM) was created using GCTA with IlluminaHD genotypes. The MLMA-LOCO analysis was run using the subset of the imputed whole genome sequence genotypes that mapped within 1 Mb either side of the peak, incorporating one covariate for ChIP-seq sequencing batch.

### Expression QTL

Reads mapping to exons in the RefSeq protein-coding gene database (AR 106 [52]) were counted for all 411 cows using the featureCounts function of the Subread software package (version 1.5.3, [65]). Genes with a median read count of less than five were excluded. The remaining expression data were aggregated by gene and processed using the Bioconductor (version 3.10, [66]) package DESeq2 (version 1.26.0, [67]), transforming the read counts using the variance-stabilising transformation (VST), to yield phenotypes suitable for mixed-model analysis. Next, outlier samples were detected and excluded using PCA on the VST-transformed phenotypes as described for ChIP data, yielding a population of 392 cows. To facilitate the discovery of exon-eQTL (eeQTL), reads were recounted on an individual exon basis, using featureCounts as described above, for a population of 371 animals that comprised the subset of the 392 cows that also had imputed genotypes available (see “Variant Calling and Genotyping” section above). Exons with median read counts of less than five were excluded. Lastly, individual exon expression phenotypes were produced by transforming the read counts using VST and adjusted to remove the effect of sequencing batch, then eeQTL were identified using the MLMA-LOCO approach implemented in GCTA, as described for ChIP-seq above, with imputed sequence genotypes extracted from within 1 Mb of the gene.

### Enrichment

Enrichment of QTL under peaks was determined using the formula outlined below [68]:

Enrichment = (C/A)/(B/D) where: A is the number of positions under peaks, B is the number of positions that were QTL, C is the number of QTL under a peak and D is the number of positions in the genome. Values greater than 1 indicate enrichment and less than 1 depletion.

### Putative causal variants

Significant dSNPs were filtered to obtain a list of putative causal variants which met the following criteria:

- *p* < 0.0001 in hQTL and eeQTL analysis and the first test of asb and ase QTL analysis.
- Located under the histone peak
- Had the same direction of effect in all 4 analyses

*P* values for each putative causal variant were calculated by identifying the Chi-square value for each individual *p*-value (from the hQTL, eeQTL, asbQTL and aseQTL analyses) and combining them. The combined *p*-value was calculated from the chi-squared distribution with 4 degrees of freedom. Only the lowest *p*-value SNP for each peak-exon pair was included. Putative causal variants described here were compared to independent publicly reported gene expression QTL from the cattle Genotype-Tissue Expression (cGTEx [25]) project. For each variant the direction of effect on each exon in our data was compared to the direction of effect for the corresponding gene for eQTL found in mammary (*n* = 175) and blood (*n* = 698). cGTEx gene eQTL data was downloaded from the cGTex website (https://cgtex.roslin.ed.ac.uk/).

### Identification of putative binding motifs

Sequence motifs were identified for asbQTL dSNPs where the variants were (a) located in the peak for which they were associated (with the exception of H3K27ac, for which very few such sites were identified), and (b) were significant at *p*-value thresholds of 1 × 10^− 8^, 1 × 10^− 7^, or 1 × 10^− 6^, for H3K4Me1, H3K4Me3, and H3K27ac respectively (based on Bonferroni, but with lower stringency where low numbers of sites were selected). Additionally, dSNPs were selected for aseQTL that were (a) within 10Kb of the TSS of their associated gene (to reduce the number of variants while enriching for *cis*-acting sites), and (b) were significant at a threshold of 1 × 10^− 15^. For all selected dSNPs, 21 bp (i.e., the SNP site ±10 bp either side) of DNA sequence was extracted from the ARS-UCD1.2 reference genome (24), as well as the corresponding reverse complement sequence, and these sequences were subsequently clustered using complete linkage with Levenshtein distances calculated between sequences measured over only their central nine bases. The resulting tree was then cut at height 3, corresponding to a maximum edit distance of three nucleotides between the central 9 bp of any pair of sequences. The resulting groups of similar sequences (excluding those with too few member sequences) were then used to produce position frequency matrices (PFMs [69];) that represent candidate transcription-factor (TF) binding motifs. Minimum set sizes to identify a motif were set to ten sequences per cluster.

The JASPAR2018 database [70] was subsequently used to identify transcription factors potentially targeting these motifs. PFMs produced by clustering were compared to those annotated for TF binding sites using the TFBSTools package (version 1.24.0 [71];) in R (version 3.6.2) using the “PFMSimilarity” function and candidate factors selected with relScore > 90%. Both the CORE and POLII collections from JASPAR2018 were used, with the CORE database limited to vertebrate taxa. Clustering and motif identification were similarly applied to the list of variants highlighted as putative causative variants (described above), with the exception that these variants were not required to be within 10Kb of the corresponding gene.

Skewedness of allele frequency towards the reference allele and the positive effect allele was investigated for each cluster. The sequences from which each cluster was produced were identified, and a list produced of the reference and positive effect direction alleles of each of the variants around which those sequences were extracted. Within each list of alleles, the numbers of each base present were compared against a null distribution of equal representation (i.e., 25% each) using the multinomial log-likelihood test, implemented in the R package XNomial (v1.04 [72]).

Enrichment of sequence motifs was explored by comparing observed and expected numbers of motif consensus sequences. Consensus sequences were produced using the following method. First, entropy scores were calculated for each base in the PFM matrices, and bases trimmed from the left and right of each matrix where the entropy (in bits) fell below 0.2. For the remaining positions (columns) in the matrix, base frequencies were sorted and summed until exceeding a threshold of 0.85, when the summed bases were incorporated into a regular expression. For example, for a column with base frequencies A = 0.4, C = 0.3, G = 0.2, T = 0.1, 0.4 + 0.3 + 0.2 > 0.85, so the text “[ACG]” would be appended. Observed genomic motif counts were then produced using Perl (v5.26.1) by matching the resulting regular expression against the sequence of each autosome, plus chromosome X, and the results summed. Expected counts were produced by first counting the number of each nucleotide in the reference sequence for the same set of chromosomes, to produce base frequencies. These were then summed for each position in the consensus sequence (e.g., for the example above, f(A) + f(C) + f(G)), then multiplied together and by the total length of sequence. Enrichment scores were calculated as the observed counts divided by the expected counts.

### Analysis of key lactation genes

We manually evaluated the gene lists presented through the ‘Putative causal variants’ analysis described above to identify candidates of prior interest. This analysis was performed in an ad hoc manner, where the aim was not to conduct a systematic survey of the literature but rather identify candidate genes that we ( [24, 27, 28, 31, 32, 35]) and others ( [26, 29, 30, 33, 34]) have recurrently highlighted through previous lactation trait GWAS and QTL studies.

For the more in-depth analyses of the *CSF2RB* gene, data was leveraged from a previous, detailed investigation of that locus [31]. Here, association statistics were recomputed for presentation in the current study, analysing milk yield phenotypic records from 29,350 cows presented in the original Lopdell, Tiplady [31] paper. This analysis tested all imputed sequence variants within an interval that was ±50Kb of the segment containing the *CSF2RB* gene and ChIP-peaks of interest (chr5:75,228,399-75,422,505), comprising 1446 variants. Association testing was performed as previously described [31], using GCTA (version 1.91.3beta [64]) and fitting mixed linear models that omitted the *CSF2RB* host chromosome (i.e. chromosome 5) from the GRM. This GRM was identical to that previously described [31], being constructed from variants from the Illumina BovineHD SNPchip. Since the Lopdell, Tiplady [31] analysis was based on data mapped to the UMD3.1 genome (i.e. the reference assembly preceding that used for all other analyses in the paper), genotype data were positionally ‘lifted over’ to the ARS-UCD1.2 reference genome (24) using a custom script, with these transposed data displayed in the current paper.

## Supplementary Information

**Additional file 1: Supplementary Table 1.** Summary data for each ChIP-seq library. Information on the number of mapped reads, quality and number of peaks for each ChIP-seq dataset.

**Additional file 2: Supplementary Table 2.** The number of genes or exons with a heterozygous SNP (tSNP), the number of genes or exons with multiple tSNP and the number of genes or exons where the tSNP phenotypes within were significantly different (*p*<0.05). **Supplementary Table 3.** For each histone modification, the number of peaks with a heterozygous SNP (pSNP), the number of peaks with multiple pSNP and the number of peaks where the pSNP phenotypes within were significantly different (*p*<0.05).

**Additional file 3: Supplementary Table 4.** The number of peak-exon pairs with shared allele-specific QTL variants as well as the distance between peak-exon pairs and the average number of times a peak was associated with an exon and vice versa. **Supplementary Table 5.** The number of peak-exon pairs with shared traditional QTL as well as the distance between peak-exon pairs and the average number of times a peak was associated with an exon and vice versa.

**Additional file 4: Supplementary Table 6.** The frequencies of the top 10 predicted transcription factor (TF) classes from the JASPAR 2018 CORE database. **Supplementary Table 7.** The frequencies of the top six predicted promotor element classes from the JASPAR 2018 POLII database.

**Additional file 5: Supplementary Table 8.** Genes associating with the putative causal variants identified in this paper.

## Data Availability

The datasets analysed for this study are publicly available. RNA-seq data is available here: https://www.ncbi.nlm.nih.gov/sra/?term=PRJNA682457. ChIP-seq data is available here: https://www.ebi.ac.uk/ena/browser/view/PRJEB52456.
