# Supplementary material for: Genetic variation in histone modifications and gene expression identifies regulatory variants in the mammary gland of cattle
Source: BMC Genomics. 2022 Dec 8;23:815. doi: 10.1186/s12864-022-09002-9 (PMC9733386; doi:10.1186/s12864-022-09002-9)
Supplement: Supplementary file 2 — Additional file 2: Supplementary Table 2. The number of genes or exons with a heterozygous SNP (tSNP), the number of genes or exons with multiple tSNP and the number of genes or exons where the tSNP phenotypes within were significantly different (p<0.05). Supplementary Table 3. For each histone modification, the number of peaks with a heterozygous SNP (pSNP), the number of peaks with multiple pSNP and the number of peaks where the pSNP phenotypes within were significantly different (p<0.05). [file 12864_2022_9002_MOESM2_ESM.docx]

***Supplementary Table 2. The number of genes or exons with a heterozygous SNP (tSNP), the number of genes or exons with multiple tSNP and the number of genes or exons where the tSNP phenotypes within were significantly different (p<0.05).***

| **Feature** | **Number of features with tSNPs** | **Number of features with multiple tSNPs** | **Number of features where phenotype of tSNPs within were significantly different (p<0.05)** |
| --- | --- | --- | --- |
| Gene | 13,429 | 11,470 | 8,018 |
| Exon | 53,483 | 22,339 | 9,646 |

***Supplementary Table 3. For each histone modification, the number of peaks with a heterozygous SNP (pSNP), the number of peaks with multiple pSNP and the number of peaks where the pSNP phenotypes within were significantly different (p<0.05).***

| **Histone modification** | **Number of peaks with pSNPs** | **Number of peaks with multiple pSNPs** | **Number of peaks where phenotype of pSNPs within were significantly different (p<0.05)** |
| --- | --- | --- | --- |
| H3K4Me3 | 293,982 | 159,650 | 18,169 |
| H3K4Me1 | 319,655 | 214,841 | 41,119 |
| H3K27ac | 475,126 | 265,469 | 29,029 |
