# Supplementary material for: Genetic variation in histone modifications and gene expression identifies regulatory variants in the mammary gland of cattle
Source: BMC Genomics. 2022 Dec 8;23:815. doi: 10.1186/s12864-022-09002-9 (PMC9733386; doi:10.1186/s12864-022-09002-9)
Supplement: Supplementary file 3 — Additional file 3: Supplementary Table 4. The number of peak-exon pairs with shared allele-specific QTL variants as well as the distance between peak-exon pairs and the average number of times a peak was associated with an exon and vice versa. Supplementary Table 5. The number of peak-exon pairs with shared traditional QTL as well as the distance between peak-exon pairs and the average number of times a peak was associated with an exon and vice versa. [file 12864_2022_9002_MOESM3_ESM.docx]

**Supplementary Table 4. The number of peak-exon pairs with shared allele-specific QTL variants as well as the distance between peak-exon pairs and the average number of times a peak was associated with an exon and vice versa.**

|  | **Number of peak-exon pairs** | **Median distance between peak and exon** | **Peaks** | **Exons** | **Average exons per peak** | **Average peaks per exon** |
| --- | --- | --- | --- | --- | --- | --- |
| **H3K27ac** | 5,350 | 344,811 | 2,017 | 2,949 | 3.1 | 2.2 |
| **H3K4Me3** | 24,423 | 340,445 | 6,052 | 7,466 | 4.8 | 4.1 |
| **H3K4Me1** | 19,541 | 375,841 | 6,145 | 6,603 | 3.7 | 3.7 |

**Supplementary Table 5. The number of peak-exon pairs with shared traditional QTL as well as the distance between peak-exon pairs and the average number of times a peak was associated with an exon and vice versa.**

|  | **Number of peak-exon pairs** | **Median distance between peak and exon** | **Peaks** | **Exons** | **Average exons per peak** | **Average peaks per exon** |
| --- | --- | --- | --- | --- | --- | --- |
| **H3K27ac** | 26,345 | 680,185 | 3,545 | 12,751 | 7.4 | 2.1 |
| **H3K4Me3** | 122,133 | 559,577 | 9,264 | 28,378 | 13.1 | 4.3 |
| **H3K4Me1** | 154,519 | 556,980 | 10,795 | 31,938 | 13.3 | 4.8 |
