# Supplementary material for: Genetic variation in histone modifications and gene expression identifies regulatory variants in the mammary gland of cattle
Source: BMC Genomics. 2022 Dec 8;23:815. doi: 10.1186/s12864-022-09002-9 (PMC9733386; doi:10.1186/s12864-022-09002-9)
Supplement: Supplementary file 4 — Additional file 4: Supplementary Table 6. The frequencies of the top 10 predicted transcription factor (TF) classes from the JASPAR 2018 CORE database. Supplementary Table 7. The frequencies of the top six predicted promotor element classes from the JASPAR 2018 POLII database. [file 12864_2022_9002_MOESM4_ESM.docx]

**Supplementary Table 6. The frequencies of the top 10 predicted transcription factor (TF) classes from the JASPAR 2018 CORE database.**

| **TF Class** | **aseQTL** | **H3K27ac** | **H3K4Me1** | **H3K4Me3** |
| --- | --- | --- | --- | --- |
| C2H2 zinc finger factors | 83 | 27 | 88 | 81 |
| Homeo domain factors | 62 | 7 | 3 | 4 |
| Rel homology region (RHR) factors | 22 | 4 | 13 | 4 |
| Basic helix-loop-helix (bHLH) factors | 12 | 6 | 16 | 5 |
| Tryptophan cluster factors | 19 | 2 | 9 | 0 |
| C2CH THAP-type zinc finger factors | 6 | 0 | 7 | 13 |
| SMAD/NF-1 DNA-binding domain factors | 19 | 0 | 4 | 2 |
| Fork head / winged helix factors | 7 | 2 | 8 | 6 |
| Nuclear receptors with C4 zinc fingers | 12 | 2 | 8 | 0 |
| High-mobility group (HMG) domain factors | 13 | 1 | 2 | 0 |

**Supplementary Table 7. The frequencies of the top six predicted promotor element classes from the JASPAR 2018 POLII database.**

| **Promotor element** | **aseQTL** | **H3K27ac** | **H3K4Me1** | **H3K4Me3** |
| --- | --- | --- | --- | --- |
| BREd | 382 | 50 | 180 | 90 |
| DCE_S_II | 72 | 10 | 85 | 41 |
| DCE_S_I | 51 | 9 | 58 | 51 |
| INR | 80 | 12 | 48 | 8 |
| DCE_S_III | 46 | 6 | 47 | 33 |
| BREu | 11 | 0 | 8 | 19 |
